# Dynamical modeling of the cholesterol regulatory pathway with Boolean networks

**DOI:** 10.1186/1752-0509-2-99

**Published:** 2008-11-24

**Authors:** Gwenael Kervizic, Laurent Corcos

**Affiliations:** 1 Inserm U613, Faculté de Médecine, Université de Bretagne Occidentale, 29238 Brest cedex, FRANCE

## Abstract

**Background:**

Qualitative dynamics of small gene regulatory networks have been studied in quite some details both with synchronous and asynchronous analysis. However, both methods have their drawbacks: synchronous analysis leads to spurious attractors and asynchronous analysis lacks computational efficiency, which is a problem to simulate large networks. We addressed this question through the analysis of a major biosynthesis pathway. Indeed the cholesterol synthesis pathway plays a pivotal role in dislypidemia and, ultimately, in cancer through intermediates such as mevalonate, farnesyl pyrophosphate and geranyl geranyl pyrophosphate, but no dynamic model of this pathway has been proposed until now.

**Results:**

We set up a computational framework to dynamically analyze large biological networks. This framework associates a classical and computationally efficient synchronous Boolean analysis with a newly introduced method based on Markov chains, which identifies spurious cycles among the results of the synchronous simulation. Based on this method, we present here the results of the analysis of the cholesterol biosynthesis pathway and its physiological regulation by the Sterol Response Element Binding Proteins (SREBPs), as well as the modeling of the action of statins, inhibitor drugs, on this pathway. The *in silico *experiments show the blockade of the cholesterol endogenous synthesis by statins and its regulation by SREPBs, in full agreement with the known biochemical features of the pathway.

**Conclusion:**

We believe that the method described here to identify spurious cycles opens new routes to compute large and biologically relevant models, thanks to the computational efficiency of synchronous simulation.

Furthermore, to the best of our knowledge, we present here the first dynamic systems biology model of the human cholesterol pathway and several of its key regulatory control elements, hoping it would provide a good basis to perform *in silico *experiments and confront the resulting properties with published and experimental data. The model of the cholesterol pathway and its regulation, along with Boolean formulae used for simulation are available on our web site . Graphical results of the simulation are also shown online. The SBML model is available in the BioModels database  with submission ID: MODEL0568648427.

## Background

### Systems biology

Systems biology is an emerging scientific field that integrates large sets of biological data derived from experimental and computational approaches. In this new paradigm, we no longer study entities of biological systems separately, but as a whole. Hence, large data sets can be translated into sets of links representative of the interactions of species from within single or multiple pathways. In fact, elementary functions in those systems are the result of the inherent characteristics of the specific elements involved and the interactions they are engaged in within the systems [1]. In biological or biomedical matters, modeling activities are strongly linked to the nature and amount of available data on the model. Furthermore, computational studies in systems biology rely on different formalisms that are intimately connected to the level of knowledge one has of a biological system. In the present study, the cholesterol synthesis pathway, including most of its associated reactions, is analyzed to address the effect of either activators or inhibitors. Hence, blockade can be attained by targeting the HMG-CoA reductase, the rate-limiting enzyme of the mevalonate pathway, with statins, widely used hypocholesterolemic drugs. Alternatively, activation of the pathway can be triggered by Sterol Response Element Binding Proteins (SREBPs), as part of a compensatory feedback mechanism. Moreover, to better analyze this pathway including both enzymatic reactions and gene regulatory networks, we will focus on the Boolean networks formalism, particularly suitable to delineate dynamic properties from qualitative information on regulatory interactions [2,3].

### Boolean formalism for qualitative modeling and simulation

A model or simulation of a biological network is said to be qualitative when each entity of this model is represented by a variable having a finite set of possible values. We can note here that the possible values that can be taken by the variable are not necessarily linearly correlated to the concentration of the represented species. Those values represent qualitative states of the entities from the network. In the formalism of Boolean networks, the state of a species is described by a Boolean variable, which value is either 1 if the species is active (i.e. its activity is detectable, in biological terms) or 0 if inactive (its activity is undetectable). Moreover, a Boolean function allows to compute the state of a species at time *t *+ 1, knowing the states of *k *other species at time *t*. If we denote by *x*_*i *_the state of species *i *and by *b*_*i*_(*x*(*t*)) the associated Boolean function, we get the following equations for the dynamics of the Boolean network:

(1) *x*_*i*_(*t *+ 1) = *b*_*i*_(*x*(*t*)), 1 ≤ *i *≤ *n*

We can note here that the Boolean formalism allows us to model various biological systems such as gene regulatory networks and metabolic networks whose entities have very different timescales.

### Construction of a Boolean network: modeling inhibition and activation

Let us detail how inhibitions and activations should be modeled in the Boolean network formalism.

• Inhibition: if A is an enzyme that produces a compound B but can be inhibited by compound C, then the Boolean function that predicts the presence of B at time *t *+ 1 will be: B(*t *+ 1) = A(*t*) AND NOT(C(*t*))

• Activation: if A is a precursor of B and the reaction of transformation of A to B is catalyzed by enzyme C, then the Boolean function that predicts the presence of B at time *t *+ 1 will be: B(*t *+ 1) = A(*t*) AND C(*t*)

Here is a simple example with 4 genes (A, B, C, D) and the 4 following Boolean functions:

• A(*t *+ 1) = NOT (D(*t*))

• B(*t *+ 1) = NOT (A(*t*))

• C(*t *+ 1) = A(*t*) OR B(*t*)

• D(*t *+ 1) = NOT (C(*t*))

**Figure 1:**
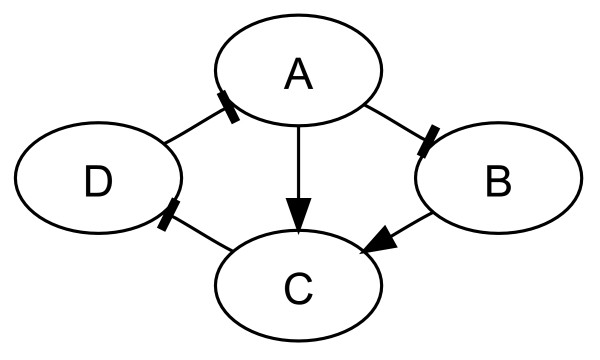
**Example of a simple regulatory network**. Graphical representation of a regulatory network with 4 genes (A, B, C, D). Its full dynamics is described in the associated Boolean functions.

The graphical representation of this network can be seen in figure 1.

### Synchronous and asynchronous paradigms in the Boolean formalism

In Boolean simulations, there are two main paradigms where conception of time and transition between states differs.

• The simplest one is the synchronous simulation. At each step of clock (time is moving discretely in Boolean simulations) all the decrease or increase calls are realized simultaneously. This approach is computationally efficient, but might lead to simulation artifacts such as spurious cycles [4,5], which are cycles that do not appear in asynchronous simulation.

• In asynchronous simulation, only one transition occurs at each clock step. Thus, the same reaction can occur several times before another one is completed, which enables the simulation of biological systems that contain slow and fast kinetics (equivalent to a stiff system in the Ordinary Differential Equations paradigm).

It is worthwhile to note that the steady states, which correspond to some phenotypes, are the same in those two paradigms. However, some dynamic behaviors can be very different.

To sum up, synchronous simulations have fewer modeling power but are more computationally efficient while asynchronous simulations are able to predict a wider range of biological behaviors but their exhaustive computation becomes intractable for large biological systems [6,7].

**Figure 2:**
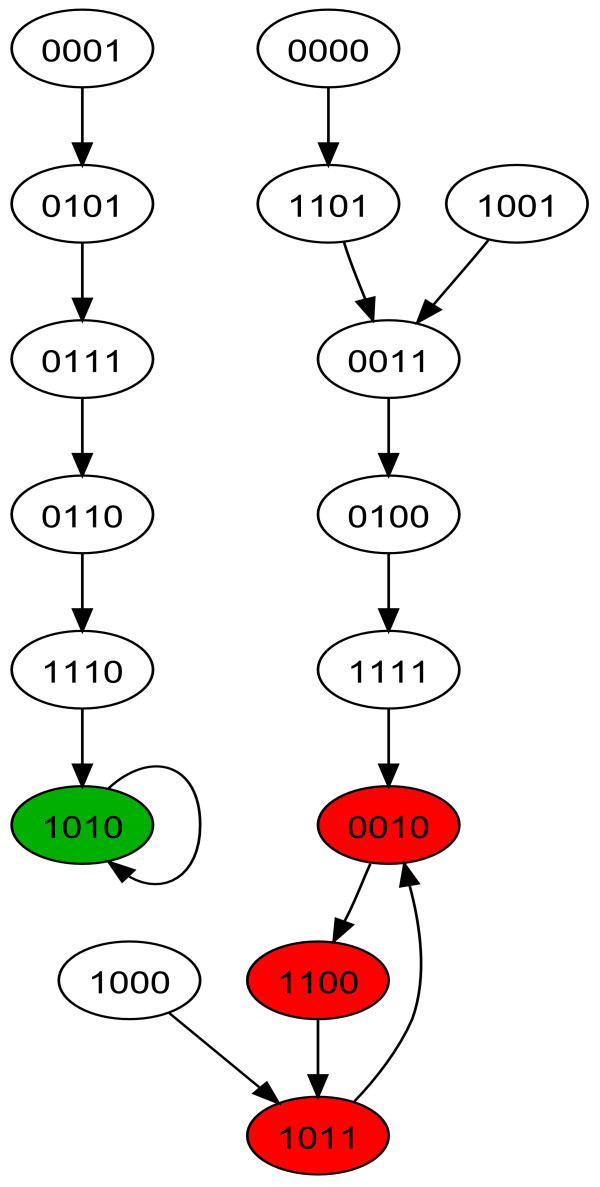
**Finite state machine of our regulatory network taken as an example in synchronous simulation**. The state [1010] colored in green corresponds to a steady state. It has 5 states and itself in its basin of attraction (i.e. the states whose trajectory during the simulation lead to this steady state). The 3 states [0010], [1100], [1011] colored in red correspond to a state cycle. They have 7 states and themselves in their basin of attraction. All the state space is shown in the figure.

In the synchronous paradigm simulation, our simple regulatory network gives the results partially shown in table 1. The study of this truth table shows that {1010} is a steady state (or point attractor, or equilibrium) and that {0010, 1100, 1011} is a state cycle (or dynamic attractor, or cyclic attractor). This becomes more evident when converting this network into a finite state machine as shown in figure 2. The state colored in green corresponds to the steady state and the states colored in red correspond to the state cycle.

**Table 1:** Fragment of the truth table obtained from our simple regulatory network.

	(ABCD)						
	
*t*	0000	0001	0010	0011	0100	0101	...
*t *+ 1	1101	0101	1100	0100	1111	0111	...

For each initial array of values (initial state) at time *t*, the new array of values, obtained by evaluating the system through the Boolean functions, is shown on the second line (*t *+ 1).

**Figure 3:**
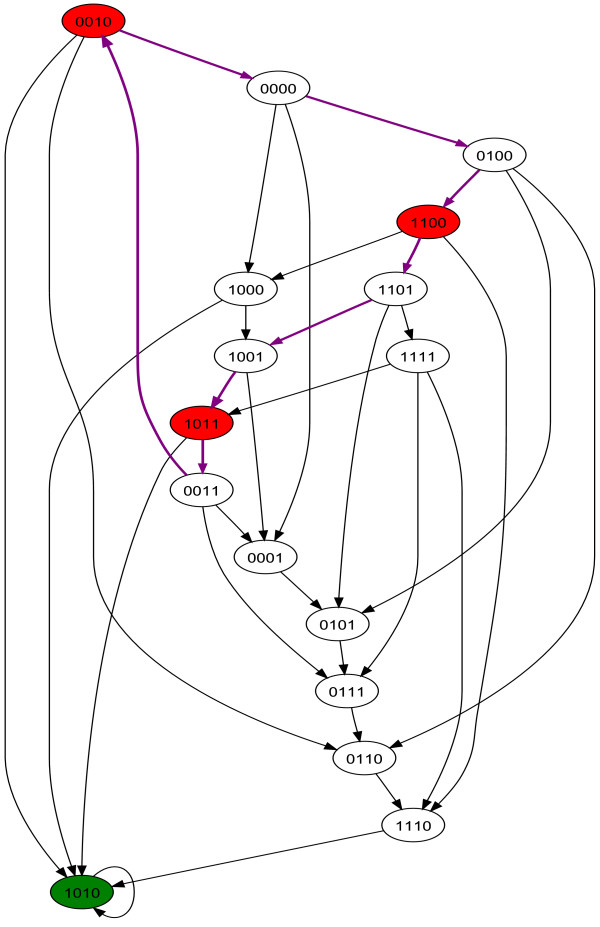
**Finite state machine of our regulatory network taken as an example in asynchronous simulation**. The state [1010] colored in green also corresponds to a steady state in the asynchronous simulation. (All the steady states are the same in synchronous and asynchronous simulations) The 3 states [0010], [1100], [1011] which correspond to a state cycle in the synchronous simulation are still colored in red, but this figure clearly shows that they do not correspond to a state cycle in the asynchronous simulation. Actually, even if there are several paths enabling to reach cyclically those three states (one of those paths is indicated with purple arrows), there are also several paths leading to the steady state from which there is no path back to those 3 states.

In the asynchronous paradigm simulation, our simple regulatory network gives the finite state machine shown in figure 3. The state colored in green corresponds to the steady state in both synchronous and asynchronous simulations. We recall that steady states are obviously always the same in synchronous and asynchronous simulations. The states colored in red are the states which correspond to the state cycle in synchronous simulation. The purple arrows propose one way -among many possible- to reach cyclically those states. Note that, for some regulatory networks, it is not possible in asynchronous simulation to reach cyclically states which form a state cycle in synchronous simulation. This becomes obvious when looking at the synchronous and asynchronous simulation results of a simple negative feedback loop of size 3. The synchronous state cycle {010, 101} does not exist in asynchronous simulation. These results are shown on our web site . Furthermore, we can have an intuition that this purple trajectory is in some way unstable because while cycling through the 3 states {0010, 1100, 1011}, the system could have gone through many transitions that lead to the steady state 1010.

### The cholesterol biosynthesis pathway

Cholesterol is an important constituent of mammalian cell membranes. It maintains their fluidity and allows other molecules playing important biological roles, like glycoproteins, to anchor to the membrane compartment. It is also the precursor of fat-soluble vitamins, including vitamins A, D, E and K and of various steroids hormones, such as cortisol, aldosterone, progesterone, the various estrogens and testosterone. It comes for about one third from the dietary intake and for about two thirds from endogenous synthesis from unburned food metabolites. Its synthesis starts from acetyl CoA, through what is often called the HMG-CoA reductase pathway. It occurs in many cells and tissues, but with higher rates in the intestines, adrenal glands, reproductive organs and liver. Cholesterol synthesis is orchestrated by a protein complex formed by the Sterol Regulatory Element Binding Protein (SREBP), the SREBP-cleavage activating protein (SCAP) and the insulin-induced gene 1 (Insig) [8-10]. This complex is maintained in a repressed state located in the endoplasmic reticulum (ER). When the cholesterol level is low, Insig1 interaction with SREBP-SCAP complex is relieved allowing SREBP-SCAP to migrate to the Golgi apparatus where SREBP is cleaved by two proteases called S1P and S2P. Once SREBP is matured, it migrates to the nucleus and acts as a transcription factor upon binding to sterol regulatory elements (SRE) to activate the genes coding for the main enzymes of the HMG-CoA reductase pathway (e.g. HMG-CoA synthase, HMG-CoA reductase, FPP synthase, CYP51). The synthesis of cholesterol can be regulated by drugs such as HMG-CoA reductase inhibitors, among which the most potent belong to the statins family [11-14]. They lower cholesterol by inhibiting the enzyme HMG-CoA reductase, which is rate-limiting.

### Effects of statins on cancer activated pathways

Therefore statins are known lipopenic drugs, but they are also drug candidates against cancer [15]. Intermediate molecules in the HMG-CoA reductase pathway undergo important biochemical reactions of prenylation whose blocking will inactivate several intracellular transduction pathways that involve Ras, Rho and small G proteins [16-19]. Hence, statins can block Ras activation, which occurs in 30% of human tumours. Experimentally, statins can stop cell growth by blocking cells at the G1 or G2/M stages, or induce apoptosis in several cancer cell types [20]. Important results have also been obtained using rodent models where neuroblastoma, colic cancer and melanoma have regressed under the effect of lovastatin. Moreover, the combination of classical antineoplastic drugs, like DNA topoisomerase inhibitors, and statins increases tumour cell killing [21,22].

In this paper, we first focus on the synchronous formalism enabling us to compute our large model of cholesterol regulatory pathway. We next propose a methodology based on asynchronous formalism and of Markov chains to overcome one of its limitations: the appearance of spurious cycles.

## Methods

### Boolean modeling of the human cholesterol regulatory pathway

**Figure 4:**
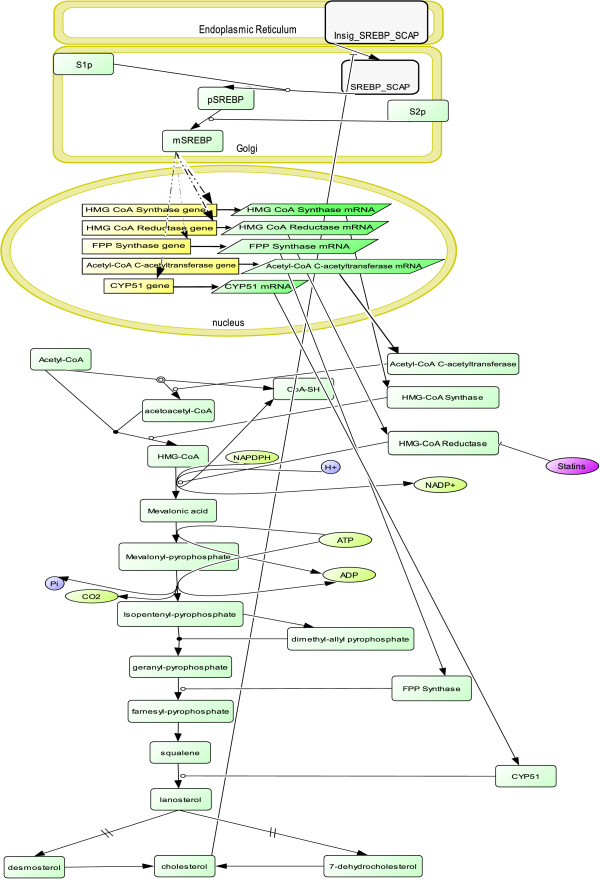
**Cholesterol Regulatory Pathway**. The cholesterol biosynthesis pathway is shown on this figure starting by its precursor Acetyl-CoA. Several of the key enzymes of this pathway regulated by SREBPs are also shown. The genetic regulation initiated by the effect of cholesterol on the Insig-SREBP-SCAP complex can be seen on the top of this figure. The action of statins, widely used hypolipidemic drugs, on the HMG-CoA-Reductase enzyme is also captured in this graph. Species of the model are grouped into cellular compartments as nucleus, golgi apparatus and endoplasmic reticulum. Default cellular compartment is the cytosol. This graphical representation has been prepared with CellDesigner [60,61].

The model shown in figure 4 has been made using data from the literature [23-25]. It is composed of the cholesterol synthesis pathway and its regulation by SREBPs.

After a few simplifications aimed at reducing the state space size, our model includes 33 species (genes, mRNAs, proteins, biochemical intermediates and statins). In particular, we assume the SREBP-SCAP-Insig1 complex to be always present in the membranes of the endoplasmic reticulum (ER). Therefore, in our model, the SREBP-SCAP complex can be either present in the ER if the cholesterol level is high, or absent if the cholesterol level is below the physiologically relevant threshold, a situation that occurs following its dissociation from Insig. We also assume that S1p and S2p are always present in the membranes of the Golgi apparatus. Hence, in our model, a non-fully matured SREBP protein, called here pSREBP (for precursor-SREBP) is automatically produced if SREBP-SCAP is present. Likewise, matured SREBP, called mSREBP, is automatically derived from pSREBP and then migrates to the nucleus to enhance transcription of the genes from the HMG-CoA reductase pathway when cholesterol levels are perceived as insufficient.

Target genes will then be transcribed into their respective mRNA, which will be translated into the corresponding enzymes. The endogenous cholesterol synthesis starts with acetyl-CoA which can, in our model, be either present, or absent in case of deficiency. Acetyl-CoA combines with itself to give CoA-SH and acetoacetyl-CoA. Acetyl-CoA reacts then with acetoacetyl-CoA to give HMG-CoA (3-Hydroxy-3-methylglutaryl CoA). This reaction is catalyzed by the HMG-CoA synthase. Therefore, the Boolean formula that describes the evolution of HMG-CoA is:

HMG_CoA(t+1) = Acetoacetyl_CoA(t) AND Acetyl_CoA(t) AND HMG_CoA_Synthase(t)

as expressed in the formalism of equation (1).

We assume that NADPH and H+ are always present and we have chosen not to represent them in our model. Thus, in the presence of HMG-CoA reductase, HMG-CoA will produce mevalonic acid. ATP is also considered to be present in sufficient amounts so that mevalonic acid will transform into mevalonyl pyrophosphate, which will then transform into isopentenyl pyrophosphate.

Isopentenyl pyrophosphate will give dimethyl allyl pyrophosphate, and then combine with its own product to form geranyl pyrophosphate. This last one will combine with isopentenyl pyrophosphate to give farnesyl pyrophosphate. Farnesyl pyrophosphate will lose two inorganic phosphates and one H+ ion to give presqualene pyrophosphate that will get two hydrogens from NADPH and H+ and lose two more inorganic phosphates to transform into squalene.

Since we assume NAPDH and H+ to be always present in enough quantity, farnesyl pyrophosphate will automatically give squalene and presqualene pyrophosphate which, as an intermediate of the reaction, is not mentioned.

The ring closure of squalene produces lanosterol. We have then omitted several transitions and jumped from the lanosterol to desmosterol or 7-dehydrocholesterol, which both give cholesterol. The Boolean formula that describes the formation of cholesterol from either desmosterol or 7-dehydrocholesterol is:

Cholesterol(t+1) = Desmosterol(t) OR 7_dehydrocholesterol(t)

Perturbations of the model such as blockade of the HMG-CoA reductase by statins, widely used hypocholesterolemic drugs, can be readily modeled. In the case of statins and HMG-CoA reductase, the Boolean formula is:

HMG_CoA_Reductase(t+1) = HMG_CoA_Reductase_RNA(t) AND NOT(Statins(t))

### Encoding the model

At each time of the simulation the state of the targeted biological system is represented by a Boolean vector. Each coordinate represents a species in the pathway.

The evolution function takes a Boolean vector representing the state of the model at time t and returns a Boolean vector representing the state of the model at time t + 1.

### Storing the model

The study of those pathways is greatly facilitated when the models are stored in a computer-readable format allowing representation of a biological system. Such formats have already been proposed like the Systems Biology Markup Language (SBML) [26,27]. SBML files are static representations of biological systems that contain species, reactions, kinetic laws and possible annotations. SBML implements the XML (Extensible Markup Language) standard and is now internationally supported and widely used. It allows models of biological systems to be stored in public or private databases. However, it has been designed for the purpose of ODE (Ordinary Differential Equations) simulations and thus needs some adaptations to be used for Boolean simulations, like the possibility to store Boolean formulae. For our purposes, we have used SBML files with additional <*BooleanLaws*> </*BooleanLaws*> tags which are stored within reaction tags in an annotated section to remain compatible with SBML standard. This SBML can be downloaded from the BioModels database [28] and has the following submission ID: MODEL0568648427.

### Simulation: point and cyclic attractors

In a synchronous simulation, every trajectory converges to an attractor. Indeed, as the state space is finite (its size is 2^*N *^with *N *the number of species in the model), if we keep the simulation running long enough it will eventually come back to an already reached state. At that point, the trajectory becomes periodic because the simulation is deterministic on a finite state space.

If the periodic part of the trajectory is of size one, we call the state a point attractor. When the system loops infinitely through several states, we call the set of these states a cycle attractor.

While non-attractor states are transient and visited at most once on any network trajectory, states within an attractor cycle or point are reached infinitely often. Thus, attractors are often identified with phenotypes [2,3]. Considering that a phenotype is an observable state, therefore stable, of an organism or a cell, real biological systems are typically assumed to have short attractor cycles [29].

### The state-space explosion problem

In order to fully analyze the model with a simple simulation approach, we would need to simulate every state of the state-space. But the size of this space grows exponentially with the number of species and thus the computation of the trajectories starting from all possible states will rapidly become too costly. Thus, we have decided, as a first step prior to a formal analysis, to use a random generator in order to choose a subset of start states significantly smaller than the whole state-space and uniformly distributed in this space. We have also taken advantage of multiple processors computing in order to cover the maximum of the state space with a minimum of time. Algorithms developed for Boolean simulation are very well suited for parallel execution. For example the set of start states used for simulation can easily be divided into subsets and simulation can be run independently from those start subsets. However, we think that parallelization is not sufficient to overcome the combinatorial explosion. Indeed, in order to add a species to a model, the computing capabilities must be multiplied by two.

### Relative importance of cycles: A Markovian approach based on asynchronous perturbations

We propose here a methodology based on Markov processes that computes the stability of cycles. Markov processes have already been used for controlling and analyzing gene networks [30,31]. Our approach differs from what is done in Probabilistic Boolean Networks (PBN) by the choice of the state space: instead of classically using the state space of the system itself (i.e. the 2^*N *^possible values of the state vector), we will use the set of state cycles and equilibria which is typically much smaller [2,3]. This allows us to compute Markov chain-based algorithms on large biological systems and thus to take into account the substantial and still growing amount of data we have on those networks and pathways. However, as a price for scalability and unlike PBN, the framework of synchronous Boolean networks does not represent the possible stochasticity of state transitions. Let S be the set of results of our Boolean analysis (S is composed of point attractors and cyclic attractors). S is the state-space of the finite discrete time-homogeneous Markov chain we want to study. In order to define the transitions of this Markov chain, we apply a perturbation on each cycle C in S. C has *k *states (*k *can be 1 in the case of a point attractor), say (*s*_1_, *s*_2_, ..., *s*_*k*_). For each state *s*_*i *_of the cycle (*s*_1_, *s*_2_, ..., *s*_*k*_) the perturbation consists in reevaluating each species by its own Boolean function triggered asynchronously. Thus we obtain *N *new states (*s*_*i*,1_, *s*_*i*,2_, ..., *s*_*i*,*N*_) for each state in C. When we perturb every state with every perturbation, we obtain a new set of perturbed states of size *kN*.

We can note here that some of the states in (*s*_*i*,1_, *s*_*i*,2_, ..., *s*_*i*,*N*_) are equal to the perturbed state *s*_*i*_. Those states will be taken into account similarly to the states different from *s*_*i*_. We then simulate synchronously all the states in (*s*_*i*,1_, *s*_*i*,2_, ..., *s*_*i*,*N*_) until they reach one of the attractors of the system (i.e. an element of S). The transition probability from a cycle to another is defined as the ratio of the number of perturbed states of the first cycle that reach the second one over the total number of perturbed states of the first cycle. We also take into account the transitions from a cycle to itself. By this mean, we add some asynchronous dynamics in our synchronous analysis. We can then compute the stationary distribution of this Markov process and interpret it as a measure of the relative importance of each cycle. We achieve this computation with an initial vector of size n and of value [1/*n*, 1*/n*, ..., 1/*n*] where n is the size of S. By this mean, we make sure that all the absorbing states are taken into consideration. The aim of this methodology is to provide a measure of the stability of each synchronous attractor, using an asynchronous perturbation. Expecting that all the resulting attractors of synchronous Boolean analysis are biologically relevant would mean that all the biochemical reactions in the network happened simultaneously. Yet we know that it is not the case. Furthermore, it is well known that, within a biological pathway, some reactions are triggered with higher frequency than others (that is why, in the ODE paradigm, a lot of systems are stiff). Thus, Boolean analysis should, in some way, take into consideration the asynchronous character of biochemical reactions. Under this assumption, asynchronous perturbations seem a logical and convenient way to provide a hint of the biological relevance of the state cycles found.

## Results

### Dynamical synchronous analysis of the model

Based on the simulations obtained from 10^5 ^random states (out of 2^33 ^≃ 8.6 × 10^9 ^possible states), we predict 3 equilibria or steady states:

• one with the complex SREBP-SCAP-Insig1 activated but a lack of precursor (Acetyl-CoA) preventing cholesterol synthesis;

• one with the presence of cholesterol precursor (Acetyl-CoA), but also the presence of statins blocking the cholesterol synthesis by inhibiting the HMG-CoA reductase enzyme;

• and one with a lack of precursor and the presence of statins.

Furthermore, we found 4 state cycles corresponding to the physiological regulation of cholesterol synthesis: when the cholesterol level is too low (equivalent to the absence of cholesterol in a Boolean formalism) there is activation of the SREBP-SCAP complex and (enhancement of the) production of all the enzymes of the cholesterol synthesis regulated by SREBP. Then, the endogenous synthesis of cholesterol starts again and when its level becomes too high (equivalent to the presence of cholesterol in a Boolean formalism) it inhibits the release of the SREBP-SCAP complex and thus the production of the above enzymes.

Among those 4 cycles one has size 29 (named cycle_0 further in this article) and the others have size 33. In the cycle of size 29 the cholesterol changes from false to true (i.e. the cholesterol gets above the threshold indicative of the activation of its synthesis by the complex SREBP-SCAP-Insig) only once per cycle, while in the cycles of size 33, the cholesterol becomes true 5 times per cycle.

### Results verification through a formal analysis using a SAT solver

The results detailed in the previous paragraph are obtained using a start space for the simulation around 10^5 ^times smaller than the state space. This method has the advantage to quickly provide some attractors for the biological system. However, when using a sample of the whole state space, there is no assurance of finding all the system attractors. Formal analysis is one way to ensure that all the attractors have been found with a computational cost that could be lower than the cost of performing simulation on the whole state space. We decided to perform such a formal analysis by running a SAT solver on our Boolean network. We recall here that the Boolean satisfiability problem (commonly called SAT-problem) [32] determines if there is a set of variables for which a given Boolean formula can be evaluated to TRUE and identifies this precise set if existing. This is an *NP-complete *problem for which some instance solvers have been developed. To achieve this formal analysis, we wrote our system of Boolean equations into a suitable dimacs file format [33] (some dimacs files used for simulation can be downloaded at ). In that way, we were able to confirm that the only attractors of size 1, 29 and 33 were those detected by our simulation tool with a random start space of size 10^5^.

### Why did we need to go further: detection of spurious cycles

The simulation performed with our model results in 4 state cycles. We believe that all those cycles do not correspond to a phenotype. These outcomes of different simulations, which are not biologically relevant, are typical of the synchronous Boolean paradigm, and are called spurious cycles [4,5]. Therefore, there is a need to measure the relative importance of the cycles found using the previous methods.

### Markov chains-based stability analysis of the previous synchronous simulation

**Figure 5:**
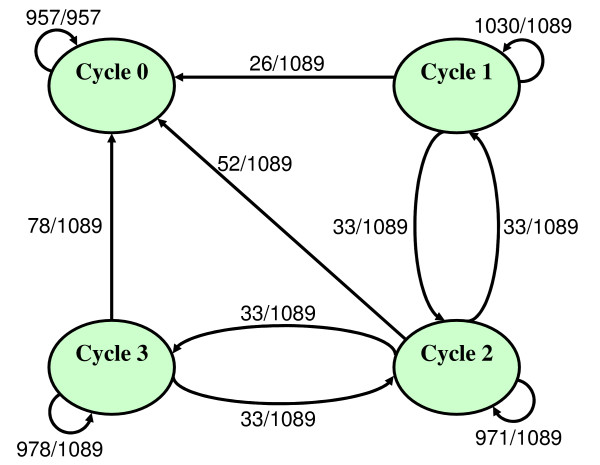
**Markov chain of the transition probabilities between state cycles in the cholesterol regulatory pathway**. Let *k *be the number of states within an attractor (*k *can be 1 in the case of a point attractor) and *N *be the number of species in the model. For each attractor of this finite time-homogeneous Markov chain, we perturb each species of each state by triggering its own Boolean function asynchronously. Thus there are *kN *perturbations per attractor. In the cholesterol regulatory pathway, one cyclic attractor found by the synchronous analysis has 29 states and the three other cyclic attractors have 33 states. The number of species in the model is 33. The weight of the edge from an attractor *X *to an attractor *Y *is the ratio between the number of perturbations of *X *which lead to *Y *over the total number of perturbations of *X*.

Let us use our stability analysis on the results of the synchronous simulation of the cholesterol regulatory pathway. We perturb the cyclic attractors found during this simulation and then simulate synchronously the states resulting from the perturbation. We interpret the ratio of the number of perturbed states of a given cyclic attractor that reach a second cyclic attractor over the total number of perturbed states of the given cyclic attractor as a transition probability. Afterwards, we use all the computed transition probabilities obtained previously to build the Markov chain shown in figure 5.

The stationary probability vector is [1, 0, 0, 0]. This reflects the fact that the state cycle 0 is absorbing (i.e. it has no outgoing transitions). We interpret this result as state cycles 1, 2 and 3 are spurious.

### Markov chains-based stability analysis of simple regulatory networks

Furthermore, to validate our method of Markov chains-based stability analysis, we applied it on simple positive and negative regulatory loops of different sizes, which are well known to contain spurious cycles. In the example of a negative feedback loop of size 3, the state cycle {010, 101} is found in synchronous simulation but does not exist in asynchronous simulation. It is obviously spurious, as detected by our method. All the detailed results and graphs can be found on our web site: .

**Figure 6:**
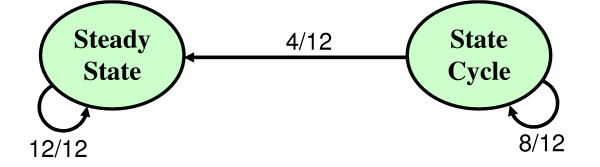
**Markov chain of the transition probabilities between the steady state and the state cycle in our simple regulatory network**. Let *k *be the number of states within an attractor (*k *can be 1 in the case of a point attractor) and *N *be the number of species in the model. For each attractor of this finite time-homogeneous Markov chain, we perturb each species of each state by triggering its own Boolean function asynchronously. Thus there are *kN *perturbations per attractor. In our simple regulatory network the cyclic attractor has 3 states. The number of species in the model is 4. The weight of the edge from an attractor *X *to an attractor *Y *is the ratio between the number of perturbations of *X *which lead to *Y *over the total number of perturbations of *X*.

When we perform this analysis on our example of a simple regulatory network (figures 1, 2 and 3) we obtain the results shown in table 2. The transition probabilities associated to the simple regulatory network shown in table 1 allow us to build the Markov chain shown in figure 6. The resulting stationary distribution of the Markov chain shown in figure 6 is [1, 0]. Thus we interpret the state cycle of our simple regulatory network as spurious.

**Table 2:** Computation of the transition probabilities associated to our simple regulatory network.

Attractors	Steady State (SS)	State Cycle (SC)		
States	1010	0010	1100	1011

Perturbed states and their limit cycles	1010 → SS	1010 → SS	1100 → SC	0011 → SC
		0110 → SS	1000 → SC	1011 → SC
		0000 → SC	1110 → SS	1011 → SC
		0010 → SC	1101 → SC	1010 → SS

Resulting probability transition	P(SS → SS) = 1	P(SC → SC) = 8/12 ≃ 0.67		
		P(SC → SS) = 4/12 ≃ 0.33		

This table shows both the principle of our newly introduced stability analysis and its application on the simple regulatory network shown in figure 1. It has 2 main columns: the steady state column and the state cycle column. As the state cycle found during the dynamical synchronous analysis contains 3 states (see figure 2), the last column is divided into 3 sub-columns. In the line "Perturbed states and their limit cycles" we show the perturbation results of each state of each attractor by re-evaluating each species by its own Boolean function triggered asynchronously. Perturbations are done from species A to species D. There are 4 species in our simple regulatory model, therefore 4 new states are generated from 1 perturbed state. We then synchronously simulate each new state and note if their simulation leads to the attractor they are derived from or to the other attractor of the system. In other words, we watch in which basin of attraction are those new states (see figure 2). The arrows following by "SC" (state cycle) or "SS" (steady state) give those responses. For the steady state no perturbation has an effect because a steady state in synchronous analysis remains a steady state in asynchronous analysis. Thus we simplify the presentation, showing that all the perturbations applied to state [1010] leave this state unchanged.

### Benchmarking of our method

The lack of a common file format for qualitative analysis is an important issue for the benchmark of such methods. This is clearly stated in the article about the SQUAD software by Di Cara et al [34]: "*With the increase of published signaling networks, it will be possible in the future to realize a benchmark among these software packages to compare their strengths and weaknesses. For doing that, however, it would be very useful to develop a common file format*." Since Di Cara's article, this issue of a common file format for qualitative analysis is still important because the current SBML format cannot encode logical models. However, work is ongoing to extend SBML such that version 3 could support information for qualitative simulation. The solution we have found to overcome this current limitation was to use a proprietary annotation with a specific namespace to remain compatible with the SBML standard. This allowed us to read and write annotated SBML files from our computational tool. However, other software cannot use our annotated SBML to perform the qualitative analysis of a biological network, unless specific code is developed to read our annotations. SQUAD can generate SBML, but it cannot use its own generated SBML to perform qualitative analysis. All the information concerning qualitative analysis is stored only in MML files (the file format used by SQUAD). This is why we could not use the current SBML version to perform the benchmark.

Furthermore, except for some well-known problems which have been well formulated and thus accepted by the community (like the test for Initial Value Problems (IVPs) solvers of the Bari University [35] or the ISCAS89 benchmark for circuits [36]), any type of benchmark would be partial and its results could be seen as unfair by other authors. For example, as mentioned by Naldi et al. in [37], some methods are only suited for a subset of biological problems: "*Garg et al. have already represented Boolean state transition graphs in terms of BDD. They considered the particular case of networks where genes are expressed provided all their inhibitors are absent and at least one of their activators is present *" [7]. The Naldi et al.'s method, regardless of its computational efficiency, is a more powerful modeling tool thanks to the use of logical evolution rules and multi-valued species. Even if it is always possible to use Boolean formalism to model multi-valued networks (by leveraging the number of Boolean species by the number of wished values, e.g. {Boolean_species_A_low_level, Boolean_species_A_middle_level, Boolean_species_A_high_level}), the use of multi-valued logical networks greatly eases the modeling process.

However, despite all the restrictions discussed above, we believe that benchmarking our method is an important issue. We have then developed a program that generates a random network whose size is a user input. For the sake of simplicity, the obtained network contains species that can have 0, 1, 2 or 3 species influencing it. This means that, to compute the state of a species at time *t *+ 1, we only need to know the state of a maximum of 3 other species at time *t*. Our benchmarking tool has 3 parameters that can modify the connection density of the network :

• the probability of being a "source" (i.e. the probability for a species to be influenced by no other species in the network),

• the probability to be under the influence of only one other species in the network,

• the probability to be under the influence of exactly two other species in the network.

The complementary probability is the probability for a species to be influenced by three other species in the network.

Then our benchmarking tool generates the files describing this network for our software as well as in MML and GINML formats for SQUAD [34,38] and GINsim [39,40], respectively.

We have analyzed networks of sizes ranging from 33 (the number of species in our cholesterol regulatory pathway) up to 2500 species.

The CPU times obtained with our method on a *Intel^® ^Core*™ *2 Duo E6600 *processor (2.4 GHz) with 2 GBytes of RAM are shown in table 3. The performance of other tested software did not compare favorably with our application. With GINsim, we were able to simulate networks as large as 1000 species, but we obtained an "out of memory" error message for the network of 2500 species. When we used the SQUAD software, we were unable to simulate a network of 1000 species or above. It is however possible that the parameters used to build the automatically generated networks might have an impact on the results of the benchmarking. Nevertheless, under the conditions used, our application is appropriate for the analysis of large biological networks.

**Table 3:** Benchmark of our method for qualitative analysis of biological networks.

number of species	33	100	250	500	1000	2500
CPU time	7.515s	15.874s	39.999s	90.295s	173.107s	569.511s

This table shows the benchmarking results of our qualitative analysis methods on automatically generated networks. Its has been done on a *Intel*^® ^*Core*™ *2 Duo E6600 *processor (2.4 GHz) with 2 GBytes of RAM.

## Discussion

The results reported here are in accordance with the biological knowledge we have on the cholesterol biosynthesis pathway. The steady states found correspond to either a lack of precursor (Acetyl-CoA) or arise from the effect of statins blocking the endogenous synthesis of cholesterol, and the cyclic attractor corresponds to a physiological regulation of cholesterol synthesis. Based on these data, we will be able to evaluate the putative impacts of additional modifications along the pathway. For instance, we may evaluate the effect of compensatory intermediates such as farnesyl pyrophosphate or geranylgeranyl pyrophosphate, which are expected to both restore cholesterol synthesis and prevent the deleterious effects of its absence [25,41-44]. These compounds may compensate for the lack of mevalonate, a condition that could readily be introduced in our computerized scheme and assayed experimentally at the same time. This would be particularly relevant in the field of cancer research, for defects in lipid signalling are of primary importance [19,45-47]. Hence, secondary protein modifications, including farnesylation or geranyl geranylation, which depend on the availability of farnesyl and geranyl geranyl pyrophosphate, respectively, are known to play pivotal roles in the progression of tumours that depend on Ras functional status (for review see [15]). However, it would require further studies to integrate our cholesterol regulatory pathway with oncological pathways, like the Ras activation pathway. We believe that this would be a particularly interesting perspective, bearing in mind that signal transduction pathways with G proteins have been extensively studied [48-52] and modeling efforts have already been made [53,54]. Furthermore, the method described here to identify spurious cycles opens new routes to compute large and biologically relevant models thanks to the computational efficiency of synchronous simulation. An important aspect was to benchmark our method in order to determine if its computational efficiency is comparable to those of GINsim and SQUAD. Our results show that our method can analyze networks containing as many as 2500 species and was time efficient. Indeed, the approach could well be applied to other regulatory pathways, either from other metabolic routes or from transduction signaling. However, the current model is purely a Boolean model where a gene is either active or inactive, a protein either present or not. An obvious limitation of Boolean formalism comes, for example, from the difficulty or the impossibility to model a simultaneous and antagonist influence on a species, e.g. if a gene is under the influence of a silencer and an activator. In that case, we would like to be able to model a threshold above which there is activation or inhibition of the targeted species, e.g. there is RNA production when there is at least twice as much activator as silencer. Boolean formalism is not suitable for this purpose. This limitation could however be alleviated by expressing the presence of a molecular species with an enumeration of values ranging from the complete lack to a highly over-expressed level such as in the generalized logical modeling approach of Thomas and D'Ari [4]. This would also enable to address, with a more realistic approach, the effect of an inhibitor or the effect of an enzyme, and to predict the preponderance of one or the other species in case of antagonistic regulation. The multi-level approach was successfully applied to many experimentally studied biological regulatory networks (e.g. [55-58]). We can note here, that our Markov chains-based stability analysis could readily be extended on the analysis of a multilevel qualitative simulation. Other work seems to be ongoing on cholesterol on cholesterol modeling using a set of ordinary differential equations thanks to a huge effort of identification of biochemical kinetics and this should add further insights on the understanding of this pathway [59]. Those two last approaches would allow us, for example, to analyze different cholesterol levels.

## Conclusion

To the best of our knowledge, this is the first description of a dynamic systems biology model of the human cholesterol pathway and several of its key regulatory control elements. This study was designed with a formal methodology and was challenged through the use of an important biochemical pathway. To efficiently analyze this model and ensure further analysis even after its complexification and possible merge with other pathway models like Ras signaling cascade models, we associate a classical and computationally efficient synchronous Boolean analysis with a newly introduced method based on Markov chains, which identifies spurious cycles among the results of the synchronous analysis. The *in silico *experiments show the blockade of the cholesterol endogenous synthesis by statins and its regulation by SREPBs, in full agreement with the known biochemical features of the pathway. Furthermore, because high throughput experiments give rise to increased complexification of biological systems, there are major needs for new computational developments for their dynamical analysis. Our methodology is one answer to this new challenge.

## Authors' contributions

LC and GK conceived this study and built the model based on literature data. GK conducted the *in silico *experiments.
